# Supplementary material for: A thermosensitive PCNA allele underlies an ataxia-telangiectasia-like disorder
Source: J Biol Chem. 2023 Mar 27;299(5):104656. doi: 10.1016/j.jbc.2023.104656 (PMC10165274; doi:10.1016/j.jbc.2023.104656)
Supplement: Supplemental Tables [file mmc1.docx]

SUPPLEMENTAL TABLES

A thermosensitive PCNA allele underlies an Ataxia Telangiectasia-like disorder

Joseph Magrino^1^, Veridiana Munford^2^, Davi Jardim Martins^2^, Thais K Homma^3,4^, Brendan Page^1,9^, Christl Gaubitz^1, 10^, Bruna L Freire^3,4^, Antonio M Lerario^4,5^, Juliana Brandstetter Vilar^2^, Antonio Amorin^6^ , Emília K E Leão^8^, Fernando Kok^6,7^, Carlos F M Menck^2^, Alexander A L Jorge^3^, Brian A Kelch^1^

**SUPPLEMENTAL TABLES**

**Table S1: Rare homozygous variants present across the three patients**

**Table S2. C148S substitution in-silico pathogenic score**

**Table S3: Data collection and refinement statistics of PCNA-C148S**

**Table S4: ITC statistics**

**Table S5. Unfolding statistics.**

**TableS-1**

**Table S1:** Rare homozygous variants present across the three patients

| **Patient 1** | | | | | | | | | | | | | | | | | | | | | | |
| --- | --- | --- | --- | --- | --- | --- | --- | --- | --- | --- | --- | --- | --- | --- | --- | --- | --- | --- | --- | --- | --- | --- |
| Chr | | Start | | End | | | Ref | | | Alt | | | | Gene | | | Effect on protein | Nucleotide Aminoacid change | | | | |
| 1 | | 152189055 | | 152189055 | | | G | | | C | | | | HRNR | | | Nonsynonymous | c.5050C>G:p.Arg1684Gly | | | | |
| 1 | | 201179497 | | 201179497 | | | G | | | A | | | | IGFN1 | | | Nonsynonymous | c.5476A>G:p.Glu1826 Lys | | | | |
| 3 | | 195510335 | | 195510335 | | | G | | | A | | | | MUC4 | | | Nonsynonymous | c.8116T>C:p.Pro2706Ser | | | | |
| 4 | | 9270413 | | 9270413 | | | T | | | G | | | | USP17L20 | | | Nonsynonymous | c.1069G>T:p.Ser357Ala | | | | |
| 4 | | 9270417 | | 9270417 | | | G | | | C | | | | USP17L20 | | | Nonsynonymous | c.1073C>G:p.Ser358Thr | | | | |
| 5 | | 43280542 | | 43280542 | | | C | | | T | | | | NIM1K | | | Nonsynonymous | c.1022T>C:p.Pro341Leu | | | | |
| 5 | | 96333752 | | 96333752 | | | T | | | C | | | | LNPEP | | | Nonsynonymous | c.1556C>T:p.Met519Thr | | | | |
| 6 | | 30587330 | | 30587330 | | | C | | | T | | | | MRPS18B | | | Nonsynonymous | c.139T>C:p.Pro47Ser | | | | |
| 6 | | 31000073 | | 31000074 | | | AA | | | - | | | | MUC22 | | | frameshift deletion | c.4770_4771del:p.Gly1590fs | | | | |
| 6 | | 36931287 | | 36931287 | | | C | | | A | | | | PI16 | | | Nonsynonymous | c.1169A>C:p.Thr390Lys | | | | |
| 8 | | 10469839 | | 10469839 | | | T | | | G | | | | RP1L1 | | | Nonsynonymous | c.1769C>A:p. Gln 590Prp | | | | |
| 13 | | 50062612 | | 50062612 | | | G | | | A | | | | SETDB2 | | | Nonsynonymous | c.1799A>G:p.Arg600Gln | | | | |
| 17 | | 25973609 | | 25973609 | | | G | | | A | | | | LGALS9 | | | Nonsynonymous | c.664A>G:p.Ala222Thr | | | | |
| **20** | | **5098255** | | **5098255** | | | **C** | | | **G** | | | | **PCNA** | | | **Nonsynonymous** | **c.443G>C:p.Cys148Ser** | | | | |
| 20 | | 37580715 | | 37580715 | | | C | | | T | | | | FAM83D | | | Nonsynonymous | c.1310T>C:p.Ser437Phe | | | | |
| **Patient 2** | | | | | | | | | | | | | | | | | | | | | | |
| 4 | | | 5990115 | | | 5990115 | C | | | | T | | | | C4orf50 | | Nonsynonymous | | c.1384G>A:p.Ala462Thr | | | |
| 11 | | | 6633369 | | | 6633369 | C | | | | T | | | | TAF10 | | Nonsynonymous | | c.52G>A:p.Ala18Thr | | | |
| 12 | | | 122958592 | | | 122958592 | G | | | | A | | | | ZCCHC8 | | Nonsynonymous | | c.862C>T:p.Arg288Trp | | | |
| 12 | | | 123109191 | | | 123109191 | G | | | | A | | | | KNTC1 | | Nonsynonymous | | c.6562G>A:p.Gly2188Arg | | | |
| 16 | | | 27221781 | | | 27221781 | G | | | | T | | | | KDM8 | | Nonsynonymous | | c.337G>T:p.Ala113Ser | | | |
| **20** | | | **5098255** | | | **5098255** | **C** | | | | **G** | | | | **PCNA** | | **Nonsynonymous** | | **c.443G>C:p.Cys148Ser** | | | |
| **Patient 3** | | | | | | | | | | | | | | | | | | | | | | |
| 9 | 43625382 | | | | 43625382 | G | | A | | | | SPATA31A6 | | | | Nonsynonymous | | c.C3305T:p.Pro1102Leu | |  |  |  |
| 9 | 43627428 | | | | 43627428 | G | | A | | | | SPATA31A6 | | | | Nonsynonymous | | c.C1259T:p.Pro420Leu | |  |  |  |
| 10 | 47000004 | | | | 47000004 | T | | C | | | | GPRIN2 | | | | Nonsynonymous | | c.T1124C:p.Val375Ala | |  |  |  |
| 10 | 51568378 | | | | 51568378 | T | | G | | | | NCOA4 | | | | Nonsynonymous | | c.T22G:p.phe8Val | |  |  |  |
| 10 | 51623190 | | | | 51623190 | T | | C | | | | TIMM23 | | | | Nonsynonymous | | c.A25G:p.Asn9Asp | |  |  |  |
| 16 | 21848694 | | | | 21848694 | T | | A | | | | NPIPB4 | | | | Nonsynonymous | | c.A1014T:p.Lys338Asn | |  |  |  |
| **20** | **5098255** | | | | **5098255** | **C** | | **G** | | | | **PCNA** | | | | **Nonsynonymous** | | **c.443G>C:p.Cys148Ser** | |  |  |  |
| 21 | 10920098 | | | 10920098 | | T | C | | | | TPTE | | | | Nonsynonymous | | | | c.A1156G:p.Lys386Glu | |  |  |

**TableS-2**

**Table S2.** C148S substitution in-silico pathogenic score

| Program | Score | Cutoff | Prediction |
| --- | --- | --- | --- |
| SIFT | 0.03 | < 0.05 | Deleterious |
| PolyPhen2 (HumVar) | 0.888 | > 0.5 | Possibly Deleterious |
| PROVEAN | -7.69 | ≤ -2.5 | Deleterious |
| CADD | 26.7 | < 15 | Deleterious |
| Mutation Assessor | 3.81 | > 0.5 | Deleterious |
| REVEL | 0.91 | ≥ 0.6 | Deleterious |

**TableS-3**

**Table S3:** Data collection and refinement statistics of PCNA-C148S

| **Data Collection** | **PCNA-C148S** |
| --- | --- |
| Space Group | C 1 2 1 |
| Wavelength | 1.54 |
| Resolution range  Unit cell dimensions | 34.97 - 3.10 |
| a, b, c (Å) | 137.64, 80.87, 70.05 |
| α, β, γ (°) | 90.00, 117.52, 90.00 |
| No. of total reflections | 30,621 (2,294) |
| No of unique reflections | 11,754 (1,059) |
| Multiplicity | 2.6 (2.2) |
| Completeness (%) | 93.0% (83.4%) |
| Mean L/σI | 6.9 (1.5) |
| Wilson B factor | 42.81 |
| R_merge_ | 0.136 (0.608) |
| R_meas_ | 0.169 (0.782) |
| R_pim_ | 0.099 (0.485) |
| CC_1/2_ | 0.983 (0.604) |
| **Refinement** |  |
| Resolution | 3.1 Å |
| R_work_ /R_free_ % | 25.52 / 29.21 |
| RMSD |  |
| Bonds (Å) | 0.003 |
| Bond angles (°) | 0.764 |
| Ramachandran favored % | 97.92 |
| Ramachandran outliers % | 0.00 |
| Rotamer outliers% | 0.00 |
| Clashscore | 10.54 |
| Average B factor | 40.13 |

**TableS-4**

**Table S4:** ITC statistics

| Protein | Ligand |  | *K_D_* (µM) | *ΔG* (kcal/mol) |
| --- | --- | --- | --- | --- |
| PCNA-WT | P21^CIP^ |  | 0.21 ± 0.04 | -9.3 ± 0.10 |
| PCNA-C148S | P21^CIP^ |  | 0.22 ± 0.07 | -9.3 ± 0.20 |
| PCNA-WT | FEN1 |  | 11 ± 2.0 | -6.8 ± 0.10 |
| PCNA-C148S | FEN1 |  | 11 ± 2.0 | -6.7 ± 0.20 |
| PCNA-WT | RNaseH2B |  | 9.7 ± 0.3 | -6.9 ± 0.03 |
| PCNA-C148S | RNaseH2B |  | 7.8 ± 0.01 | -7.0 ± 0.01 |
| PCNA-WT | P66 |  | 3.0 ± 0.16 | -7.6 ± 0.05 |
| PCNA-C148S | P66 |  | 2.8 ± 0.33 | -7.7 ± 0.07 |
| PCNA-S228I | P66 |  | 4.0 ± 0.14 | -7.5 ± 0.02 |

**TableS-5**

| Table S5. Unfolding statistics. | | | | | | | |  |
| --- | --- | --- | --- | --- | --- | --- | --- | --- |
| Protein | *T_m_* (Cº) | *ΔG* (kcal/mol) | | *ΔH (kcal/mol)* | *m* (kcal/mol/[D]) | *C_m_ (*M Gdm-HCl) | |  |
| PCNA-WT | 52.0 ± 0.03 | 8.20 ± 0.04 | 150 ± 3. | | 3.21 ± 0.01 | 2.55 | |  |
| PCNA-C148S | 42.0 ± 0.06 | 7.59 ± 0.60 | 111 ± 3 | | 3.73 ± 0.33 | 2.03 | |  |
| PCNA-S228I | 44.0 ± 0.10 | - | 103.6 ± 5 | | - | | - |  |
