# Supplementary material for: A thermosensitive PCNA allele underlies an ataxia-telangiectasia-like disorder
Source: J Biol Chem. 2023 Mar 27;299(5):104656. doi: 10.1016/j.jbc.2023.104656 (PMC10165274; doi:10.1016/j.jbc.2023.104656)
Supplement: Supplemental Text [file mmc2.docx]

SUPPLEMENTAL TEXT

A thermosensitive PCNA allele underlies an Ataxia Telangiectasia-like disorder

Joseph Magrino^1^, Veridiana Munford^2^, Davi Jardim Martins^2^, Thais K Homma^3,4^, Brendan Page^1,9^, Christl Gaubitz^1, 10^, Bruna L Freire^3,4^, Antonio M Lerario^4,5^, Juliana Brandstetter Vilar^2^, Antonio Amorin^6^ , Emília K E Leão^8^, Fernando Kok^6,7^, Carlos F M Menck^2^, Alexander A L Jorge^3^, Brian A Kelch^1^

SUPPLEMENTAL TEXT
Detail Case reports

**Detailed Patient Case reports.**

Patient 1: The index case is a boy born and initially referred to the clinic due to short stature at the age of 2.9 years. He was born to healthy, consanguineous parents with normal stature who had a history of spontaneous abortions. Intrauterine growth retardation was noted, and he was born preterm (30.5 weeks) with a birth weight of 1,000 g [-1.5 standard deviation score (SDS)], birth length of 33 cm (-2.8 SDS), and head circumference of 26.5 cm (SDS= -1.1). At birth, the patient was diagnosed with coarctation of the aorta and patent ductus arteriosus, which was corrected surgically at 15 days of age and again at 2.0 years old. After the second cardiac surgery, he had a spinal cord injury which resulted in temporary motor and sensory damage. Additionally, he failed to thrive and hit neurodevelopmental milestones. Physical examination showed mild dysmorphisms: a broad forehead, low-set posteriorly rotated ears, broad nasal bridge, thin upper lip, and cutaneous telangiectasia on the face (Figure 2C-F). In addition to his physical abnormalities, he began showing photosensitivity at two years of age. His neurological exam showed dysphonia and diffuse moderate hypotonia, and he could not sit without support. His myotatic reflexes were present, symmetric and norm intense, except for the absent bilateral ankle reflex. The Babinski sign is absent. At the first evaluation, the patient was severely short with a height SDS of -6.8 and sitting height/height (SDS= +3.3); body mass index (BMI) (SDS= -4.8), and microcephaly (head circumference SDS = -4.1).

Patient 2: Patient two is a girl who started being evaluated at 10.9 years of age. She was born at full term, small for gestational age [birth weight of 2,300 g (SDS= -2.3), birth length of 45 cm (SDS = -2.0)]. Her parents are healthy, consanguineous (first cousins), with normal height (mid parental height SDS= -1.0) and had one prior spontaneous abortion. Clinically, patient two was normal until she was 2.5 years old. At that time, it was noted that she had a gait instability that progressed until she was a wheelchair user age five. She was evaluated by a neurologist, who diagnosed her with moderate dysarthria, diffuse mild hypotonia, dystonic posture on feet (varus bilateral), without spasticity, and mild axial ataxia. The neurologist also noticed that she could assume orthostasis, with limbs in extension and slightly flexing the knees. Her physical examination showed oval palate, ocular and cutaneous telangiectasia, photophobia, and photosensitivity. The patient had recurrent respiratory infections with frequent need for antibiotic use. Laboratory investigation disclosed a slight deficiency of IgG and IgA with normal white blood cell count. The patient has severe short stature (height SDS = -4.0), body disproportion (sitting height/height SDS = +3.1), low body mass index (SDS= -2.1) and microcephaly (head circumference SDS = -2.6).

Patient 3: Patient three is an 11 years-old girl with severely short stature (height SDS = -3.6). She is the single child of a healthy and non-consanguineous couple from a small, isolated town in northeast Brazil (Supplemental Figure 1). No complications during pregnancy or childbirth were reported, and she was born to term (38.5 weeks) with a birth weight of 2.,550g (SDS= -0.4) and a birth length of 48 cm (SDS= -1.3 SDS). During her infancy, the patient was evaluated due to postnatal growth retardation, learning disability, and recurrent upper airway infections. At age four, mobility problems developed, and she had difficulty walking. She was diagnosed with dysarthria, dysphonia, dysphagia, and mild hearing loss. Her physical examination showed oval palate, down slanting palpebral fissures, small and low-set posteriorly rotated ears, nevus, ocular and cutaneous telangiectasia (Figure 2D and E), ligament laxity, scoliosis, and flat foot. A neurologist evaluated her and identified mild cognitive impairment, bradykinesia, oculomotor apraxia, and gait ataxia. Clinical laboratory studies revealed a slight deficiency of IgM, IgG, and IgA with normal white blood cell count. After starting immunoglobulin and prophylactic antibiotics, the patient’s infectious episodes reduced. She also had slight atrophy of the cerebellum (Figure 2F).
